# Isolation of equid alphaherpesvirus 3 from a horse in Iceland with equine coital exanthema

**DOI:** 10.1186/s13028-021-00572-4

**Published:** 2021-02-02

**Authors:** Lilja Thorsteinsdóttir, Gunnar Örn Guðmundsson, Höskuldur Jensson, Sigurbjörg Torsteinsdóttir, Vilhjálmur Svansson

**Affiliations:** 1 Institute for Experimental Pathology, Biomedical Centre, University of Iceland, Keldur, Keldnavegur 3, 112 Reykjavík, Iceland; 2 Ásvegur 10, Hvanneyri, 311 Borgarnes, Iceland; 3 Nautabú, Hjaltadal, 551 Sauðárkrókur, Iceland

**Keywords:** DNA polymerase gene, Glycoprotein G, PCR, Sequencing, Titration, Virus isolation

## Abstract

Equine coital exanthema (ECE) caused by equid alphaherpesvirus 3 (EHV-3) is a contagious venereal disease. It is characterized by the formation of papules, vesicles, pustules and ulcers on the external genitals of both mares and stallions. The Icelandic horse is the only breed in Iceland and has lived isolated in the country for over 1000 years. Three types of equine herpesviruses (EHV) have been found in Iceland, EHV-4, EHV-2 and EHV-5, while EHV-1 has never been detected. Symptoms resembling ECE have previous been observed in horses in Iceland, arousing suspicion of EHV-3 infection, but this has never been confirmed using virological methods. Samples were collected from a mare with papules on the vulva and inoculated in primary equine kidney cells. Cytopathic effects developed as rounded cells and syncytial formation. Polymerase chain reaction and sequencing of the partial glycoprotein G and DNA polymerase genes identified the isolated virus as EHV-3. On the basis of the findings, EHV-3 infection was verified for the first time in the native Icelandic horse population.

## Findings

Equid alphaherpesvirus 3 (EHV-3) is a highly contagious virus that causes equine coital exanthema (ECE). ECE has venereal transmission and is characterized by the formation of papules, vesicles, pustules and ulcers on the external genitals of stallions and mares. In uncomplicated cases, healing is usually completed within 10–14 days, but depigmented, cutaneous scars can persist [1]. The incubation period is usually 5–9, days but can be as short as 2 days [1, 2]. After primary infection the virus establishes a latent infection; the site of latency is unknown, but it has been hypothesized that the virus persists in the sacral ganglion as has been shown for some other venereal transmitted alphaherpesviruses [3]. Neutralizing antibodies to EHV-3 are most often found in horses of breeding age, which supports the hypothesis of transmission of the virus primarily through coitus [2]. Lesions on the lips and nostrils after non-coital transmission are also known [4] and transmission by contaminated fomites has been implicated [5]. General signs of infections, such as fever, dullness and anorexia, are sometimes more intense in stallions than in mares. Stallions with extensive ECE lesions can also exhibit loss of libido and refuse to mate with mares [2]. The virus is, however, non-invasive, and the disease relatively benign and do usually not resulting in systemic illness [1, 6].

The virus was first isolated in 1986 [2] and has worldwide distribution. EHV-3 is highly host specific and replicates almost exclusively in cells of equine origin [1]. Sequencing of the complete genome has revealed that the genome is, 151 kbp in size, has a 68% G + C content and encodes for 76 distinct genes [6]. Genetically EHV-3 is the most divergent of the alphaherpesvirues, with only 62–65% overall nucleotide homology with other EHVs [6]. In additions, the virus is serologically and antigenically different from other EHVs [1]. Veterinarians in Iceland have noted clinical signs resembling ECE, but the presence of EHV-3 has never been confirmed by proper methods. The aim of the present study was to isolate and confirm the presence of EHV-3 virus in the native Icelandic horse population.

**Fig. 1:**
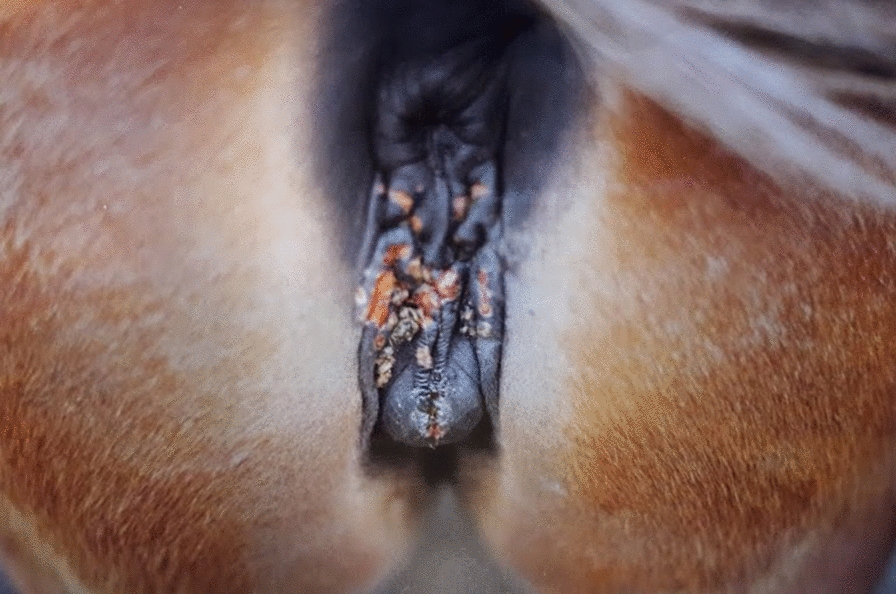
Lesions of equine coital exanthema on the vulva and perineal skin of a mare

Scrapings from ECE-like lesions were collected from a three-year-old mare with vesicles, pustules, ulcers and inflammation in the vulva mucosa and perineal skin that reached down to the epidermis (Fig. 1). The sample was stored at 4 °C overnight. The next day, 2 mL of Dulbecco’s modified eagle’s medium (DMEM, Thermo Fisher Scientific, Waltham, MA, USA) with 10% fetal bovine serum (FBS, Thermo Fisher Scientific) was added to the swab and mixed, 500 μL of the sample was inoculated onto confluent primary equine fetal kidney cells (prmEqFK) [7] at passage 10, in T25 culture flask*.* After inoculation at 37 °C for 90 min in a humidified atmosphere with 5% CO_2_, the inoculation was removed and 5 mL of DMEM supplemented with 2 mM glutamine, 100 U/mL penicillin, 100 μg streptomycin, 5% FBS) and 0.25 μg/mL Amphotericin B (Thermo Fisher Scientific) were added to the cultured cells. When most of the cells showed cytopathic effect (CPE) they were harvested, and DNA was extracted from 200 μL of supernatant with Gentra Puregene Cell Kit (Qiagen, MD, USA), according to the manufacturer´s protocol. Primers for glycoprotein G (gG) and DNA polymerase were designed using the primer3web and published GenBank sequences (GenBank accession numbers AF081188 and AF514779, respectively) (Table 1). Polymerase chain reaction (PCR) was carried out using the following cycle: 10 × Taq buffer, 2 mM dNTP, 20 μM primers, 1-unit Taq polymerase (New England Biolabs, Ipswich, MA, USA), 1 μL DNA and ddH_2_O, for total of 20 μL reaction. The cycling profile was as follows: 94 °C for 4 min, 30 cycles of 94 °C for 30 s, 54 °C for 45 s, 72 °C for 1 min and 72 °C for 7 min with final soaking at 4 °C. Agarose (Sigma-Aldrich, St. Louis, USA) was melted in 0.5 × TBE (Tris borate-EDTA, 0.045 M Tris borate and 0.001 M EDTA, pH 8.0) and 2 drops of 1:10 diluted ethidium bromide (Sigma-Aldrich) was added. The amplicons were examined after electrophoresis at 75 V for 45 min. The PCR products were extracted from the agarose gel with QIAquick Gel Extraction Kit (Qiagen) and sequenced with sanger sequencing by GeneWiz Inc. (Leipzig, Germany). EHV-3 virus stock was harvested from prmEqFK cells in passage 4, with 100% CPE. Titration with limiting dilution was done in 10-fold dilutions, 10^–1^ to 10^–10^, eight wells per dilution in 96 well plates with 25,000 cells/well, done on prmEqFK, fetal equine kidney and lung cell with extended life span (extEqFK and extEqFL, respectively) [7], rabbit kidney cells (RK13) and Vero cells. Trypsinized cells in DMEM, 50 μL, with 2–20% FBS, depending on the cell type, were incubated with 50 μL of appropriate virus dilution in DMEM, at 37 °C in humidified atmosphere with 5% CO_2_. After 4–6 h, 50 μL of DMEM was added to each well. The plated were observed daily for CPE for 7 days.

**Table 1:** Nucleotide sequence of primers used in PCR amplification and sequencing

Gene	Primers sequence 5′–3′	Location^a^	Amplicon size (bp)		GenBank no	Size of sequence strand (bp)
Glycoprotein G						
Forward	ACCACCTGCGAGACCATTAC	566–585	616		MN689934	590
Reverse	TAGTTGGTCCCCTTCTGCTG	1162–1181				
DNA polymerase						
Forward	CCCGTTGATGACCCCTATGT	822–841	321		MN689935	321
Reverse	TAGCAGCATGTCTCGCCC	1125–1142				

^a^Location starting for the first start codon, is based on EHV-3 strain, AR/2007/C3A (GenBank no NC_024771.1)

**Fig. 2:**
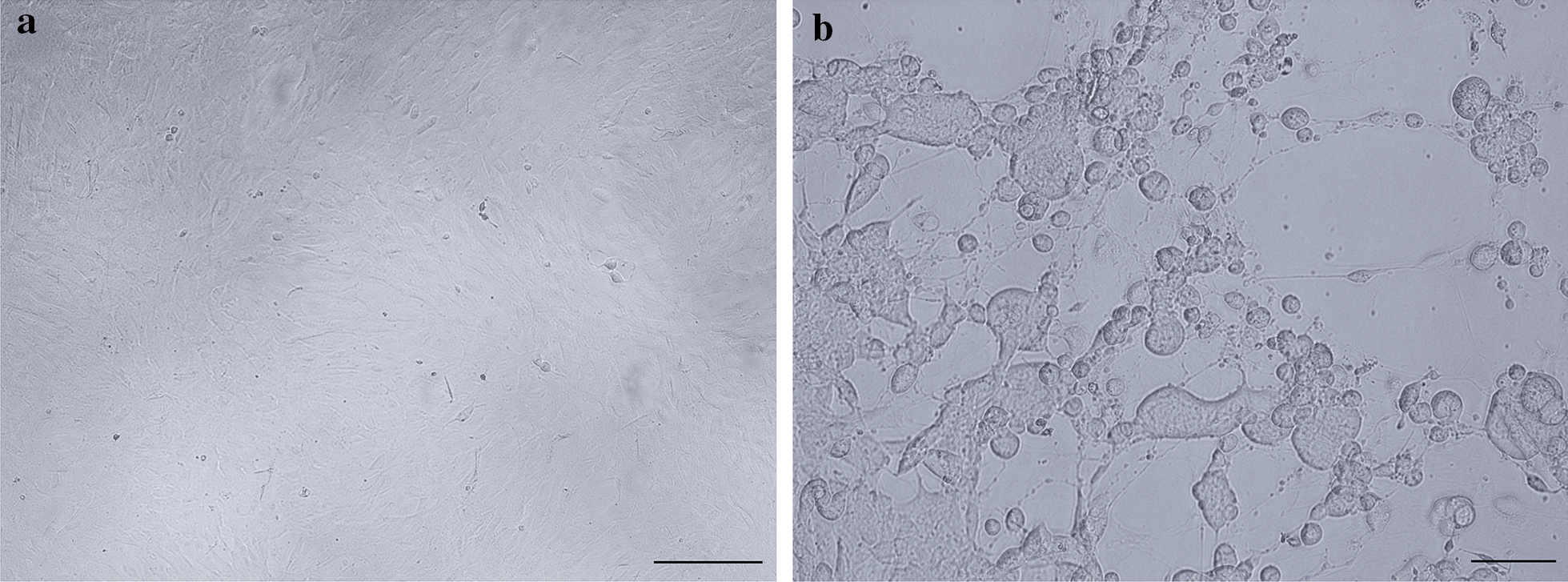
Cytopathic effect of EHV-3 in primary equine kidney cells. **a** prmEqFK cell monolayer, scale bar 20 μM. **b** EHV-3 infected prmEqFK cells, scale bar 50 μM

**Fig. 3:**
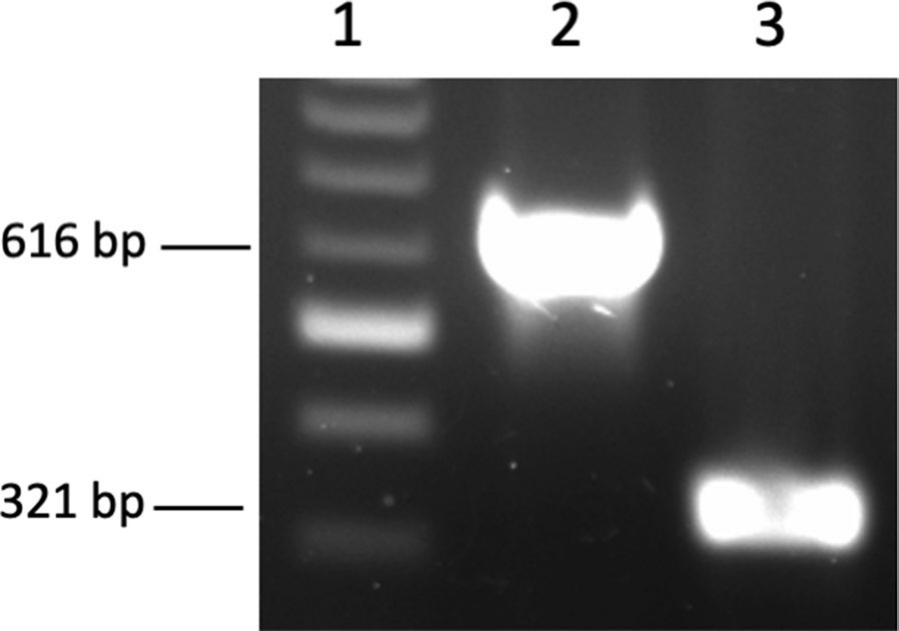
Electrophoresis on glycoprotein G and DNA polymerase gene amplicons. 1: Log2 ladder. 2: gG gene, 616 bp. 3: DNA polymerase gene, 321 bp

After virus inoculation on prmEqFK, CPE was observed after four days, characterized by rounded cells and syncytial formation (Fig. 2). Two days after the second passage the cell culture was uniformly infected. PCR amplification of both the partial gG and DNA polymerase genes gave single strong bands of the correct sizes, 616 bp and 321 bp, respectively (Fig. 3). To confirm further, the PCR products were sequenced. The sequences have been published on the NCBI database, GenBank accession numbers MN689934 and MN689935 for gG and the DNA polymerase gene, respectively. Analyzes with the NCBI Basic Local Alignment Search Tool (BLAST) program, glycoprotein G was found to have 99% nucleotide homology (588/590 identities) and the DNA polymerase gene 100% when compared to the EHV-3 strain AR/2007/C3A (GenBank accession number NC_024771.1). These results confirmed that the mare was infected with EHV-3 and expressed clinical signs of ECE.

It has been shown that EHV-3 replicates almost exclusively in cells of equine origin. We therefore tested the growth of dilutions of the EHV-3 virus stock in five different cells culture systems and determined the cytopathy with a light microscope. Three different equine fetal cells were tested, both primary cells and cells with extended life span. The primary cells can be passage about 10–12 times as compared to 40 passage for the cells with extended life span [7]. The titers were 10^5.7^, 10^5.11^ and 10^5.56^ TCID_50_/mL, respectively, for the three equine cell culture tested, prmEqFK, extEqFK and extEqFL. As expected, no CPE was observed in the RK13 and Vero cells as they are not equine cells.

The Icelandic horse is the only breed in Iceland and has been isolated from other horses for more than 1000 years [8]. Due to the isolation the horses are immunologically naïve to numerous pathogens known to infect horses in other countries. However, Icelandic horses can now be found in more than thirty countries worldwide and less than one-third of the population is living in Iceland. They are still retained as a closed population and import of horses as well as semen and embryos is prohibited by law. However, growing popularity of the breed abroad with frequent traveling of people working with Icelandic horses offers a threat the unique infectious status. Consequences of this can be seen in several introductions of new infectious equine agents in recent decades [9]. Therefore it is of importance to have an updated overview and knowledge of infectious agents already present in the population. This is the first report of EHV-3 in the native Icelandic horse population. The prevalence of EHV-3 infections in Iceland is unknown and yet to be examined, but until now infections with EHV-3 has not been considered to have severe impact on the horse breeding in Iceland.

We have previously speculated that the absence of EHV-1 infections in the Icelandic horse population might indicate that this virus was not as common in Europe in the 9th and 10th centuries as it is today [10]. The existence of ECE in the Icelandic horse breed could indicate the opposite for EHV-3, i.e. that the virus was common in horses in the medieval Europe.

## Data Availability

The datasets used and/or analyzed during the current study are available from the corresponding author on reasonable request.

## Abbreviations

CPE: Cytopathic effect
DMEM: Dulbecco’s modified eagle’s medium
ECE: Equine coital exanthema
EHV-3: Equid alphaherpesvirus 3
extEqFK: Equine fetal kidney cells with extended life span
extEqFL: Equine fetal lung cells with extended life span
FBS: Fetal bovine serum
gG: Glycoprotein G
PCR: Polymerase chain reaction
prmEqFK: Primary equine fetal kidney cells
RK13: Rabbit kidney cells
